# Supplementary material for: Impact of vaccination timing and coverage on measles near elimination dynamics: a mathematical modelling analysis
Source: Nat Commun. 2025 Sep 29;16:8601. doi: 10.1038/s41467-025-63710-w (PMC12480554; doi:10.1038/s41467-025-63710-w)
Supplement: Supplementary file 1 — Supplementary Information [file 41467_2025_63710_MOESM1_ESM.pdf]

Supplementary material

S1. Description of the case data

Data on all confirmed measles cases in England between 2010 and 2019 were collected by Public Health England (now the UK Health Security Agency). For each case, the symptom onset date, region of residence, age, and vaccine status was reported. We only included cases reported in England with no missing information on age and onset date. If the region of residency was not reported (996 cases), we used the region of the general practitioner who reported the case (95% of individuals resided in the same region as the general practitioner when both were reported). The final dataset contained 7504 cases.

Vaccine status was labelled “no” for unvaccinated cases, “v1” for individuals who had received one dose of the vaccine before being infected, or “v2” for individuals who had received two doses of the vaccine. Between 2014 and 2019, 143 individuals had their vaccine status reported as “yes”. We classified these individuals as one-dose recipients, as 1/ no other individual was classified as v1 between 2014 to 2019 (whereas 152 were classified as v2) and 2/ 19 of the individuals classified as yes were 3 years or younger, which is younger than the recommended age for MMR2 in the UK (current recommended age of vaccination: 3 years and 4 months for the second dose). Vaccine status was unknown for 105 patients (1·4% of all cases) from all age groups, regions, and outbreak years, and these cases were set as unvaccinated.

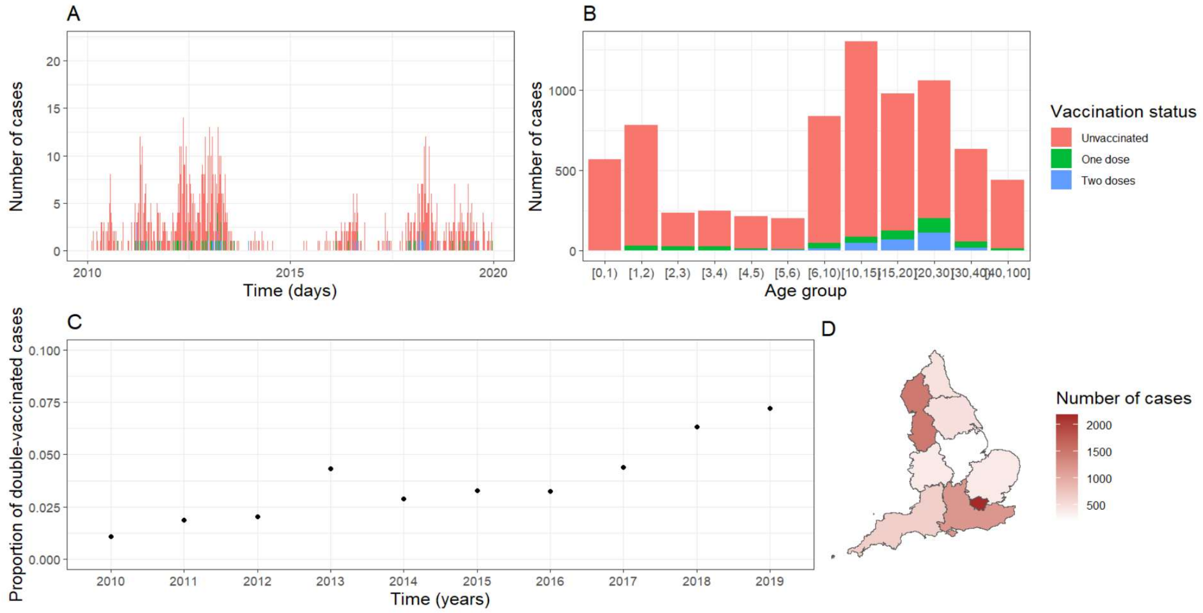

Figure S1. Case data from the surveillance dataset. A) Number of daily measles cases in England, stratified by vaccination status. B) Number of cases by age group and vaccine status. C) Proportion of double vaccinated cases each year. D) Incidence by region of England between 2010 and 2019. Reproduced from (1)

S2. Description of the vaccine coverage data

Overall, the coverage estimates for the MMR vaccine in England in CPRD are consistently between 2-4% higher than in COVER (see Figures 2& 3 and Table 1). The same applies for coverage at regional level for both and second MMR2 dose with London as the only exception which has a lower coverage for the second MMR dose in CPRD than in COVER from 2015 onwards (see Figure S3).

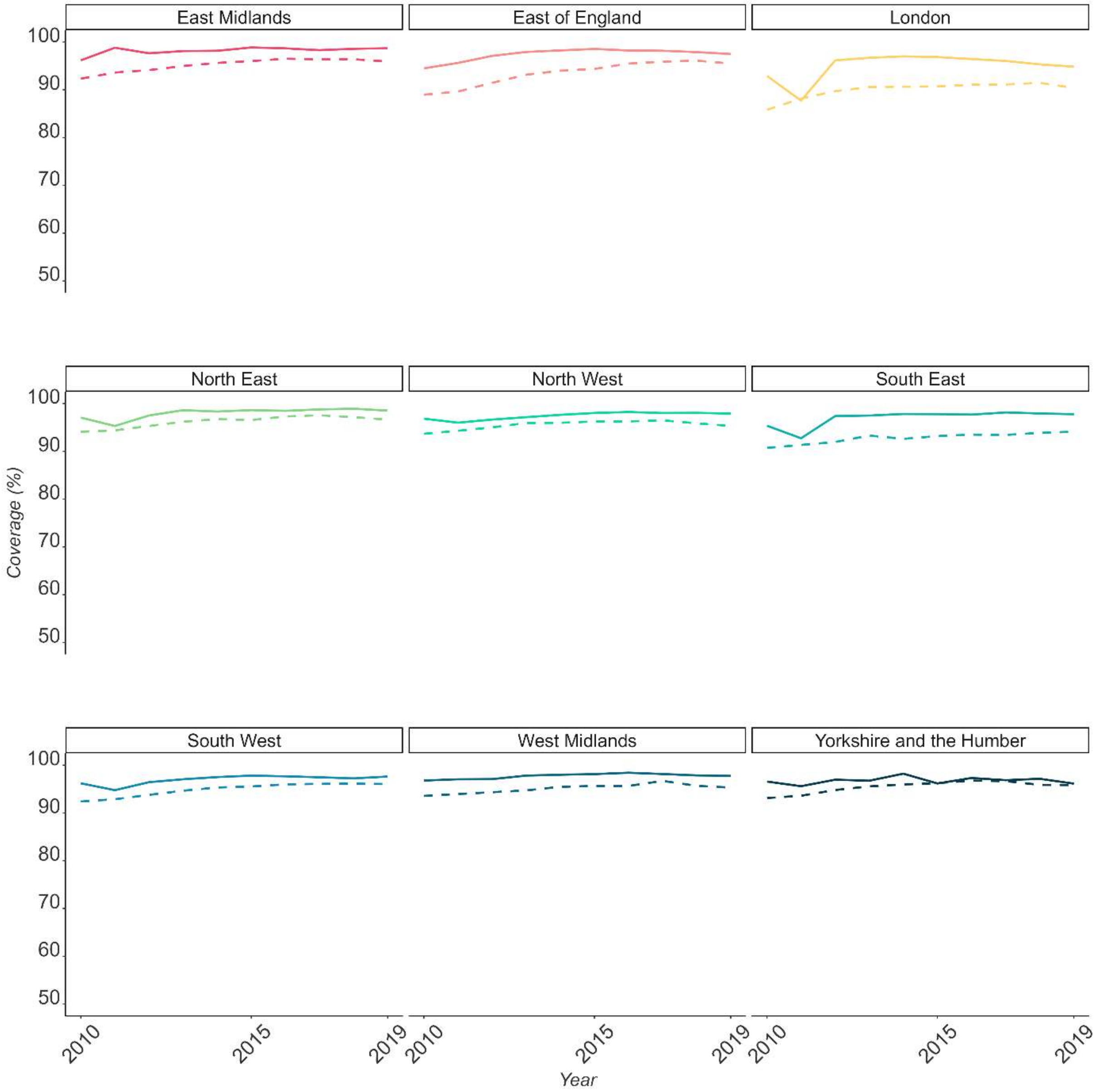

Figure S2. **Comparing vaccine coverage by data source and region** – MMR1 at the age of five in CPRD (dashed line) and COVER.

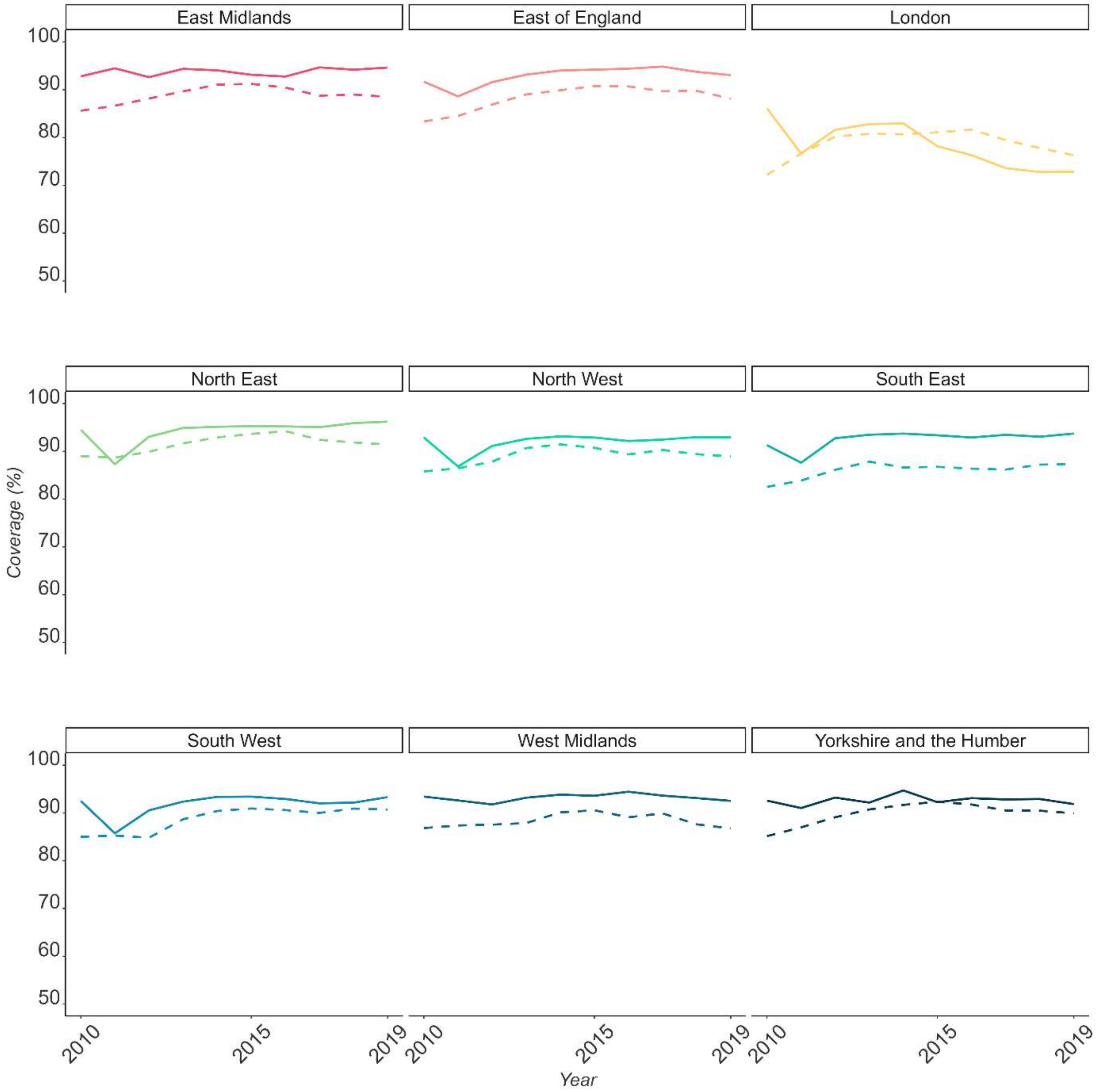

Figure S3. Comparing vaccine coverage by data source and region – MMR2 at the age of five in CPRD (dashed line) and COVER.

Table S1. Comparison vaccine coverage at age five by data source

**Year**	**MMR vaccine coverage in CPRD**	**National estimates for MMR in COVE**
2009-10	-	82.7
2010-11	-	84.2
2011-12	90.04	86.0

2012-13	90.78	87.7
2013-14	91.56	88.3
2014-15	91.23	88.6
2015-16	90.50	88.2
2016-17	89.91	87.6
2017-18	89.37	87.2
2018-19	89.20	86.4

S3. Model description

We fitted a deterministic compartmental model stratified by age and region to the case data in order to estimate the parameters of the model. The parameter sets were then used to generate stochastic simulations between Jan 1, 2010, and Dec 31, 2019, using a stochastic process to compute the number of transitions between compartments. The stochastic simulations showed the set of dynamics the parameter estimates could generate, with a higher variance than the deterministic fits. The equations of the model are fully described in Robert et al (1). The model is fully described and accessible in the following Github repository: https://github.com/alxsrobert/seirvodin/tree/main

Overview

We used a deterministic, compartmental transmission model stratified by region and age group (<1;1-2;2-3;3-4;4-5;5-6;6-10;10-15;15-20;20-30;30-40;40+ years old), implemented with the R package odin.dust (2). In each age group and region, individuals were classified in several compartments: susceptible (i.e. not vaccinated nor previously infected), vaccinated once and protected, vaccinated once but failed to seroconvert (i.e. primary vaccine failure), vaccinated twice and protected, vaccinated twice but failed to seroconvert (i.e. consecutive primary vaccine failure), exposed (E), infectious (I) and recovered (i.e. previously infected). This is illustrated in Figure S4. On infection, the individuals moved to the exposed compartment, then they become infectious and finally recover. Depending on the vaccine coverage data, a proportion of individuals gained vaccination as they age. We assumed that vaccines provide protection according to an "all or nothing" principle (3,4): while a majority of newly vaccinated individuals gain full life-long protection, a proportion of individuals do not respond to the vaccine (primary vaccine failure). This proportion is estimated by the model.

Due to the short duration of exposure and infection in comparison to the duration spent in each age strata (at least one year), there was no ageing in the exposed and infected compartments. The overall demographic structure was based on UK census data per region and simplified, ignoring migration between regions and countries and death in younger age groups.

We set the coverage of the first dose at the age of one to 75% of the coverage at age two. This was to represent that the first dose is usually given at the age of one, so children would have some protection between their first and second birthdays. Similarly, we set the coverage of the second dose at the age of three to 50% of the coverage at the age of four to account for the nine months of the fourth year of life which are spent vaccinated when the second dose is given at the age of three years and four months. This was to avoid bias in the model which would otherwise assume that there was no vaccination before age two and no second dose before age four due to the age bands in the model.

We assumed that no individuals in the 30-40 and 40+ age groups in 2010 was vaccinated, but the model estimated the proportion of individuals who gained immunity through infection.

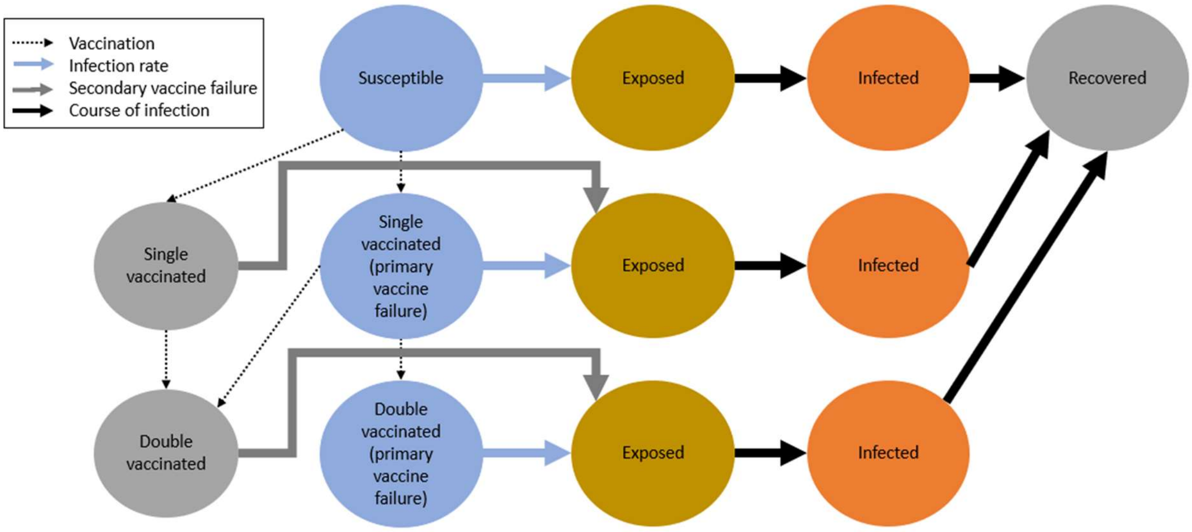

Figure S4. **Structure of the model and its compartments.** Reproduced from (1)

Contacts between age groups were included to the model based on the contact matrix from the POLYMOD study (5).

Contacts across the nine regions of England were approximated using a spatial kernel functions (see estimated parameters in table S2-S3). This kernel is a gravity model, depending on population in both regions, and distance. Distance is accounted for by the degree of connectivity between regions (neighbours have a degree of one, neighbours of neighbours have a degree of two etc.).

The within-year seasonality of transmission was estimated by two parameters (X and Y). For each time t, the infection rate (β) is computed as $\beta_t = \beta * (1 + X * cos(\frac{2*\pi*t}{365.25} + Y))$.

The average number of importations per year and region was computed using the number of cases who were classified as "imported" or "import related" in the individual case data. The daily importation rate by age group and region was then computed by dividing the local number of importations for each year by 365 and weighted by the number of inhabitants per age group. As importations may be less likely to be reported than other cases, the importation rate by region is divided by p_{import}, the report rate of imported cases. The model also estimates two parameters X_{import} and Y_{import}, to estimate the seasonality of importations in a given year.

$$n_{import}(a, i, t) = \frac{n_{import}\big(i, year(t)\big)}{365 * p_{import}} * \frac{N_{ai}}{N_i} * (1 + X_{import} * cos(\frac{2 * \pi * t}{365.25} + Yimport))$$

With N_{ai} the number of inhabitants of age a in i, N_i the number of inhabitants in region i, $n_{import}(i, year(t))$ the number of cases classified as "imported" or "import related" in the individual case data at each year.

Table S2: Summary of the model parameters

Parameter	Fixed /estimated	Value / Prior	Reference
Infection rate β	Estimated	U(0, 50)	
Duration of maternal immunity (days) δ	Estimated	N(90, 5)	(6)
Risk of onward transmission from vaccinated cases, compared to unvaccinated cases v_{onward}	Estimated	Beta(10,10)	(7)
Seasonality of infection rate: X	Estimated	U(0, 7)	
Seasonality of infection rate: Y	Estimated	U(0, 7)	
Mean duration of latent period (days) γ	Fixed	11	
Mean duration of infectious period (days) α	Fixed	8	(8–10)
Waning of immunity per year v_{leak}	Fixed in main analysis, estimated in sensitivity analysis	0, if estimated U(0,1)	
Proportion of primary vaccine failure v_{fail}	Estimated	U(0, 1)	
Risk of secondary vaccine failure v_{sec}	Fixed*	0, If estimated: U(0,1)	
Proportion of 20-30 yo individuals vaccinated during the 1996 catch-up campaigns $catchup$	Estimated	U(0, 1)	
Proportion of 6-10 yo individuals vaccinated during the 2008 catch-up campaign $catchup2$	Estimated	U(0, 1)	
Proportion of previously infected 10-15 yo $recov10{-}15$	Estimated	U(0, 1)	
Proportion of previously infected 15-20 yo $recov15{-}20$	Estimated	U(0, 1)	
Proportion of previously infected 20-30 yo $recov20{-}30$	Estimated	U(0, 1)	
Proportion of previously infected 30-40 yo $recov30{-}40$	Estimated	U(0, 1)	
Proportion of previously infected 40+ yo $recov40+$	Estimated	U(0, 1)	
Spatial parameter b	Estimated*	U(0, 5)	
Spatial parameter c	Estimated*	U(0, 5)	
Spatial parameter θ	Estimated*	U(0, 1)	

Proportion of importations reported p_{import}	Estimated	U(0, 1)	
Seasonality of importations X_{import}	Estimated	U(0, 7)	
Seasonality of importations Yi_{mport}	Estimated	U(0, 7)	

Table reproduced from supplementary material of (1)

Parameter estimates and comparison with data

Compared to the analysis presented in Robert et al (1), we updated the implementation of the models so that there is no changes to the vaccination status of individuals after 5 years of age. The distribution of vaccine status is then maintained when individuals aged into the 6-10 years old group and above. This update (and the stochasticity associated with model fitting) led to a new set of parameter estimates (Table S3), which are very close to those presented in Robert et al (Supplementary Table S3 of (1)). We then used 100 parameter sets from the deterministic model fits to generate 2,500 stochastic simulations per scenario (25 simulations per parameter set).

The comparison between the age and spatial distribution of the cases in the data and in the different simulations sets is presented in Figure S5. As discussed in Robert et al (1), the simulations using CPRD data were able to capture the overall age distribution of the cases, although the number of cases in infants were underestimated. On the other hand, the simulations using the COVER data better captured the number of cases in infants, but underestimated the number of cases in children (aged 1-2), and overestimated the number of cases in teenagers (aged 10-15 and 15-20). Only the model including waning could capture the number and age distribution of vaccinated cases (Figure S6).

Table S3: Parameter estimates in each scenario (Median and 95% credible intervals in brackets).

Parameter	Prior	CPRD data, Without waning	CPRD data, with waning	COVER data, without waning
Infection rate β	U(0, 50)	30 (29 - 32)	32 (30 - 33)	16 (15 - 17)
Duration of maternal immunity (days) δ	N(90, 5)	174 (167 - 179)	176 (171 - 180)	103 (96 – 112)
Risk of onward transmission from vaccinated cases compared to unvaccinated cases v_{onward}	Beta(10,10)	0.81 (0.68 - 0.90)	0.71 (0.55 - 0.85)	0.67 (0.48 - 0.82)
Seasonality of infection rate: X	U(0, 7)	0.16 (0.14 - 0.19)	0.16 (0.14 - 0.18)	0.17 (0.15 – 0.19)
Seasonality of infection rate: Y	U(0, 7)	5.94 (5.85 - 6.08)	5.97 (5.87 - 6.08)	6.00 (5.87 – 6.10)
Waning of immunity per year v_{leak}	U(0,1)	0	2.7 (2.3 - 3.1) e-4	0
Proportion of primary vaccine failure v_{fail}	U(0, 1)	0.05 (0.05 - 0.05)	0.02 (0.02 - 0.03)	0.07 (0.06 - 0.07)
Proportion of individuals aged 20-30 in 2010 vaccinated during the 1996 catch-up campaign $catchup$	U(0, 1)	0.85 (0.81 - 0.89)	0.66 (0.57 - 0.73)	0.80 (0.74 - 0.85)
Proportion of individuals aged 6-10 in 2010 vaccinated during the 2008 catch-up campaign $catchup2$	U(0, 1)	0.01 (0 - 0.05)	0.02 (0 - 0.06)	0.37 (0.31 – 0.43)
Proportion of previously infected 10-15 yo $recov_{10-15}$	U(0, 1)	0 (0 - 0.02)	0 (0 - 0.03)	0.01 (0 – 0.05)
Proportion of previously infected 15-20 yo $recov_{15-20}$	U(0, 1)	0.01 (0 - 0.05)	0.05 (0 - 0.11)	0.32 (0.25 – 0.39)
Proportion of previously infected 20-30 yo $recov_{20-30}$	U(0, 1)	0.43 (0.39 - 0.48)	0.51 (0.47 - 0.56)	0.49 (0.45 – 0.53)

Proportion of previously infected 30-40 yo $recov_{30-40}$	U(0, 1)	0.98 (0.98 - 0.98)	0.98 (0.98 - 0.98)	0.96 (0.96 – 0.97)
Proportion of previously infected 40+ yo $recov_{40+}$	U(0, 1)	0.99 (0.99 - 0.99)	0.99 (0.99 - 1)	0.99 (0.99 – 0.99)
Spatial parameter b	U(0, 5)	0.01 (0 - 0.02)	0.01 (0 - 0.03)	0.01 (0 – 0.04)
Spatial parameter c	U(0, 5)	0.94 (0.92 - 0.95)	0.94 (0.92 - 0.95)	0.95 (0.92 – 0.96)
Spatial parameter θ	U(0, 1)	0.93 (0.85 - 0.99)	0.94 (0.86 - 1)	0.93 (0.81 – 1)
Proportion of importations reported p_{import}	U(0, 1)	0.52 (0.49 - 0.56)	0.52 (0.48 - 0.55)	0.54 (0.50 – 0.58)
Seasonality of importations X_{import}	U(0, 7)	0.83 (0.70 - 0.96)	0.84 (0.71 - 0.97)	0.91 (0.76 – 1)
Seasonality of importations Y_{import}	U(0, 7)	4.62 (4.44 - 4.89)	4.61 (4.42 - 4.84)	4.57 (4.33 – 4.81)

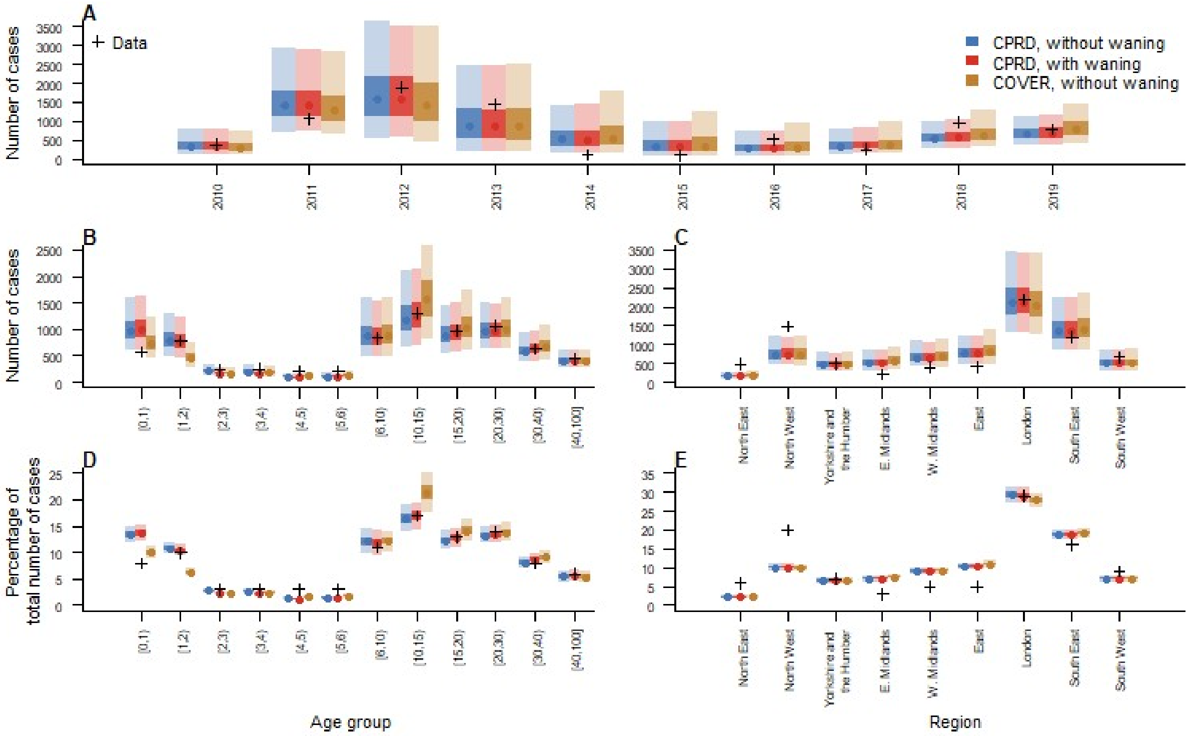

Figure S5: Estimated cases for CPRD with and without waning and COVER. *A. Number of cases per year in the reference simulation set of each scenario and in the data across all regions and age groups. B. Number of cases by age groups across all regions and years. C. Number of cases by regions across all regions and years. D. Proportion of cases by age groups. E. Proportion of cases by regions. Black crosses represents the value observed in the data, the dark area shows the 95% simulation interval, the light area shows the 50% simulation interval, the dot shows the median value in each scenario*

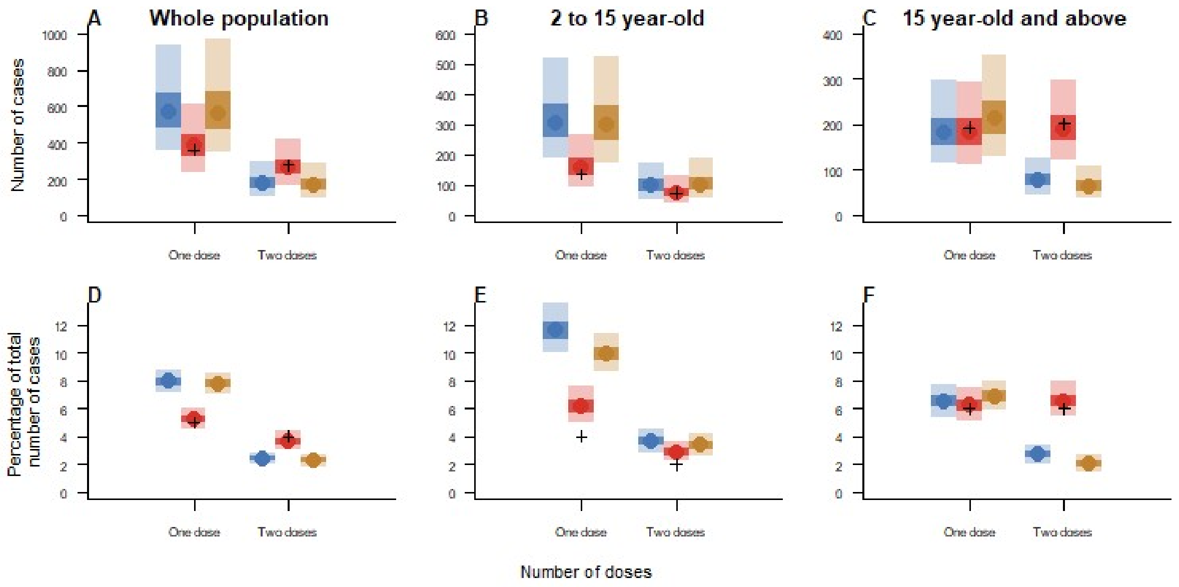

Figure S6: Distribution of vaccine status in the reference simulation set for each scenario. *A. Overall number (and D. Proportion) of vaccinated cases in each scenario. B. Number (and E. Proportion) of vaccinated cases between 5 and 15 years old. C. Number (And F. Proportion) of vaccinated cases 15 years and older. All plots show the number of cases across all regions and years. In panels B-G, black crosses represents the value observed in the data, the dark area shows the 95% simulation interval, the light area shows the 50% simulation interval, the dot shows the median value in each scenario.*

S4. Impact of vaccine schedule and coverage on transmission dynamics: reference scenario

Table S4. Number of measles cases and proportions avoided by vaccination strategy

**Vaccination Scenario**	**Median (IQR) number of cases across simulations**	**% of cases avoided in comparison to the median of the reference scenario (IQR)***
Reference	7169 (6096.5; 8534.5)	0 (-19.05; 14.96)
Scenarios of different vaccination schedules		
**MMR1 +0.25%**	6572 (5598; 7739.5)	8.33 (-7.96; 21.91)
**MMR1 +0.5%**	6116.5 (5198; 7084)	14.68 (1.19; 27.49)
**MMR1 +1%**	5134 (4467.5; 5912)	28.39 (17.53; 37.68)
**MMR2 +1%**	7086 (6002.25; 8336)	1.16 (-16.28; 16.27)
**MMR2 +3%**	6856 (5850.75; 8188)	4.37 (-14.21; 18.39)
Scenarios of different vaccination schedules		
**late MMR2**	12464 (10157.5; 15364.5)	-73.86 (-114.32; -41.69)
**early MMR2**	6318.5 (5494.75; 7377)	11.86 (-2.9; 23.35)
**early MMR2 with improved uptake +0.25%**	6359 (5461.75; 7547.25)	11.3 (-5.28; 23.81)
**Early MMR2 with improved uptake +0.5%**	6384 (5459; 7503.5)	10.95 (-4.67; 23.85)
**Early MMR2 with improved uptake +1%**	6335.5 (5356.75; 7405.25)	11.63 (-3.3; 25.28)
**Early MMR2 with coverage as MMR1**	5564.5 (4818; 6448.5)	22.38 (10.05; 32.79)
**Early MMR2 with decreased uptake -3%**	6617.5 (5655.75; 7794.25)	7.69 (-8.72; 21.11)
**Early MMR2 with decreased uptake -5%**	6882 (5836.25; 8113.5)	4.00 (-13.17; 18.59)

*A positive percentage is the percentage of cases avoided; a negative percentage is the percentage of additional cases in comparison to the median of the reference scenario. The proportion of the cases avoided in comparison to the median was calculated as follows: (Ncases-Median(reference)/Median(reference))*100.

Table S5. Number of measles cases and cases avoided by vaccination strategy stratified by year.

Year	Reference scenario	MMR1 + 0.5%		Early MMR2	
	**Median number of simulated cases (IQR)**	**Median number of simulated cases (IQR)**	**% change in comparison to the median of the reference scenario**	**Median number of simulated cases (IQR)**	**% change in comparison to the median of the reference scenario**
2010	320 (227.75; 442)	313 (224; 438)	2.19 (-36.88; 30)	298 (210; 411.25)	6.88 (-28.52; 34.38)
2011	1421 (1132.75; 1794.25)	1309 (1034; 1644.75)	7.88 (-15.75; 27.23)	1292 (1019.75; 1610.25)	9.08 (-13.32; 28.24)
2012	1600.5 (1150; 2142.25)	1321 (940; 1796)	17.46 (-12.21; 41.27)	1358.5 (965.75; 1852)	15.12 (-15.71; 39.66)
2013	845 (536.75; 1307.25)	635.5 (397; 970)	24.79 (-14.79; 53.02)	704.5 (431; 1046)	16.63 (-23.79; 48.99)
2014	504 (333; 749)	398.5 (274; 568)	20.93 (-12.7; 45.63)	445 (300; 637)	11.71 (-26.39; 40.48)
2015	313 (197; 477)	242.5 (154; 365.25)	22.52 (-16.69; 50.8)	271 (171; 404)	13.42 (-29.07; 45.37)
2016	258 (172.75; 381)	213.5 (151; 306)	17.25 (-18.6; 41.47)	232 (160; 337.25)	10.08 (-30.72; 37.98)
2017	340 (248; 455)	285 (213; 384.25)	16.18 (-13.01; 37.35)	312 (231; 415)	8.24 (-22.06; 32.06)
2018	541 (439; 671)	469 (381; 572)	13.31 (-5.73; 29.57)	508 (407; 624)	6.1 (-15.34; 24.77)
2019	663 (542.5; 801.25)	559 (461.75; 672)	15.69 (-1.36; 30.35)	608 (503; 735)	8.3 (-10.86; 24.13)

Table S6. Number of measles cases and cases avoided by vaccination strategy stratified by region.

**Region**	**Reference scenario**	**MMR1 + 0.5%**		**Early MMR2**	
	**Median number of simulated cases (IQR)**	**Median number of simulated cases (IQR)**	**% change in comparison to the median of the reference scenario**	**Median number of simulated cases (IQR)**	**% change in comparison to the median of the reference scenario**
North East	157 (132; 187)	132 (112; 156)	15.92 (0.64; 28.66)	137 (117; 162)	12.74 (-3.18; 25.48)
North West	729 (611.75; 868.25)	612 (524; 717)	16.05 (1.65; 28.12)	634 (548; 742.25)	13.03 (-1.82; 24.83)
Yorkshire and the Humber	468 (393; 553)	392 (334; 459)	16.24 (1.92; 28.63)	408 (352; 477.25)	12.82 (-1.98; 24.79)
East Midlands	504 (425; 603)	427 (360; 499)	15.28 (0.99; 28.57)	440 (377; 518)	12.7 (-2.78; 25.2)
West Midlands	655.5 (551; 779.25)	555 (468; 645)	15.33 (1.6; 28.6)	573 (493; 672)	12.59 (-2.52; 24.79)
East of England	746.5 (632; 888.25)	635 (536; 740)	14.94 (0.87; 28.2)	653 (565; 766)	12.53 (-2.61; 24.31)
London	2079 (1771; 2465)	1786 (1531.75; 2073)	14.09 (0.29; 26.32)	1859 (1614; 2164.25)	10.58 (-4.1; 22.37)
South East	1340 (1139; 1606.25)	1141 (965; 1328)	14.85 (0.9; 27.99)	1180 (1019; 1383.25)	11.94 (-3.23; 23.96)
South West	501 (423; 595.25)	422 (361; 494)	15.77 (1.4; 27.94)	439 (378; 514)	12.38 (-2.59; 24.55)

S5. Impact of vaccine schedule and coverage on transmission dynamics: COVER data scenario

Table S7. Number of measles cases and cases avoided by vaccination strategy using COVER data instead of CPRD data

Vaccination Scenario	Median (IQR) number of cases across simulations	% of cases avoided in comparison to the median of the reference scenario (IQR)*
**Reference**	7177.5 (6024.25; 8566)	0 (-19.35; 16.07)
Scenarios of different vaccination schedules		
**MMR1 +0.5%**	6367.5 (5339.75; 7482.5)	11.29 (-4.25; 25.6)
**MMR1 +1%**	5657.5 (4797.5; 6719.5)	21.18 (6.38; 33.16)
Scenarios of different vaccination schedules		
**late MMR2**	10276 (8345; 12758.75)	-43.17 (-77.76; -16.27)
**early MMR2**	6660 (5590.75; 7989.5)	7.21 (-11.31; 22.11)
**Early MMR2 with improved uptake +1%**	6603.5 (5575.25; 7919.75)	8 (-10.34; 22.32)
**Early MMR2 with improved uptake +3%**	6569 (5569.75; 7804.25)	8.48 (-8.73; 22.4)
**Early MMR2 with coverage as MMR1**	6114.5 (5164; 7282.25)	14.81 (-1.46; 28.05)
**Early MMR2 with decreased uptake -3%**	6782 (5746.75; 8114.75)	5.51 (-13.06; 19.93)
**Early MMR2 with decreased uptake -5%**	6947 (5869.5; 8332.25)	3.21 (-16.09; 18.22)

*A positive percentage is the percentage of cases avoided; a negative percentage is the percentage of additional cases in comparison to the median of the reference scenario. The proportion of the cases avoided in comparison to the median was calculated as follows: Ncases-Median(reference)/Median(reference))*100.

Table S8. Number of measles cases and cases avoided by vaccination strategy stratified by year using COVER data.

Year	Reference scenario	MMR1 + 0.5%		Early MMR2	
	**Median number of simulated cases (IQR)**	**Median number of simulated cases (IQR)**	**% change in comparison to the median of the reference scenario**	**Median number of simulated cases (IQR)**	**% change in comparison to the median of the reference scenario**
2010	299 (212; 414.25)	287 (200; 405)	4.01 (-35.45; 33.11)	284 (195; 402)	5.02 (-34.45; 34.78)
2011	1266.5 (983; 1652.5)	1208 (935; 1582.5)	4.62 (-24.95; 26.17)	1202 (947; 1568.5)	5.09 (-23.85; 25.23)
2012	1408 (980; 1955.5)	1279 (879; 1784.5)	9.16 (-26.74; 37.57)	1333 (909; 1859.25)	5.33 (-32.05; 35.44)
2013	842 (496; 1269.5)	677 (409.75; 1067.25)	19.6 (-26.75; 51.34)	746.5 (440; 1165.25)	11.34 (-38.39; 47.74)
2014	534 (333.75; 804)	425 (283; 639.25)	20.41 (-19.71; 47)	468.5 (305; 733)	12.27 (-37.27; 42.88)
2015	324.5 (191.75; 527)	261.5 (164; 430)	19.41 (-32.51; 49.46)	296 (176; 491)	8.78 (-51.31; 45.76)
2016	269 (173; 412)	235 (157; 357.25)	12.64 (-32.81; 41.64)	256 (168; 388.25)	4.83 (-44.33; 37.55)
2017	360 (258; 494.25)	316 (229; 442)	12.22 (-22.78; 36.39)	329 (236; 462.25)	8.61 (-28.4; 34.44)
2018	614.5 (482; 778)	542.5 (427; 681.25)	11.72 (-10.86; 30.51)	570 (450; 721)	7.24 (-17.33; 26.77)
2019	774 (614; 959)	660 (535.75; 817.25)	14.73 (-5.59; 30.78)	710 (573.75; 893.25)	8.27 (-15.41; 25.87)

Table S9. Number of measles cases and cases avoided by vaccination strategy stratified by region using COVER data.

Region	Reference scenario	MMR1 + 0.5%		Early MMR2	
	**Median number of simulated cases (IQR)**	**Median number of simulated cases (IQR)**	**% change in comparison to the median of the reference scenario**	**Median number of simulated cases (IQR)**	**% change in comparison to the median of the reference scenario**
North East	161 (134; 195)	142 (119; 170)	11.8 (-5.59; 26.09)	150 (125; 182)	6.83 (-13.04; 22.36)
North West	702 (585; 843)	625 (523.75; 738)	10.97 (-5.13; 25.39)	653 (548; 786)	6.98 (-11.97; 21.94)
Yorkshire and the Humber	461 (383; 552)	406.5 (339; 484)	11.82 (-4.99; 26.46)	429 (357; 512.25)	6.94 (-11.12; 22.56)
East Midlands	533.5 (447; 649)	473 (392.75; 565)	11.34 (-5.9; 26.38)	495.5 (414; 597)	7.12 (-11.9; 22.4)
West Midlands	664 (553.75; 797)	587 (491; 694)	11.6 (-4.52; 26.05)	616 (516; 741)	7.23 (-11.6; 22.29)
East of England	789 (663.75; 945)	695 (581; 822)	11.91 (-4.18; 26.36)	733 (613; 872)	7.1 (-10.52; 22.31)
London	1991 (1682; 2371.25)	1772 (1492; 2082.25)	11 (-4.58; 25.06)	1852 (1568; 2221.25)	6.98 (-11.56; 21.25)
South East	1366 (1146; 1636)	1202 (1011; 1429)	12.01 (-4.61; 25.99)	1271.5 (1061.75; 1527)	6.92 (-11.79; 22.27)
South West	500.5 (420; 602)	443 (370; 528)	11.49 (-5.49; 26.07)	465 (390; 559.25)	7.09 (-11.74; 22.08)

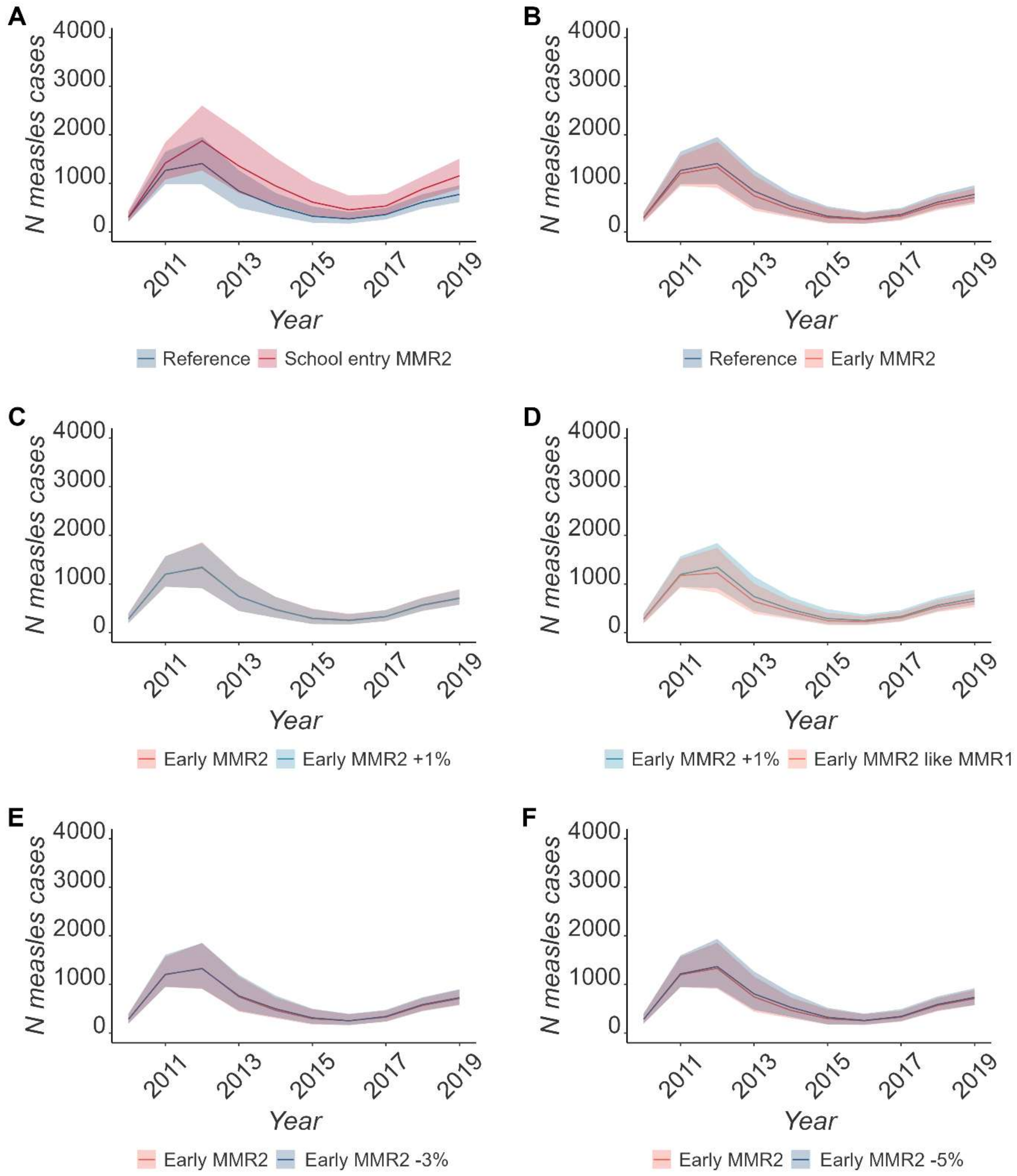

Figure S7: Comparing the reference scenario to scenarios with changes in the immunisation schedule using COVER data. Comparing the median number and IQR of simulated cases across simulations using COVER data without waning between (A) Reference scenario and MM2 given at school age, (B) Reference scenario and MMR2 given at the age of two, (C) MMR2 given at the age two against an increase of MMR1 by 1%., (D) increased MMR1 by 1% and an earlier MMR2 with the same coverage as MMR1. (E) and (F) are comparing the early MMR2 with the same uptake as before against a drop in coverage by 3% and 5% respectively.

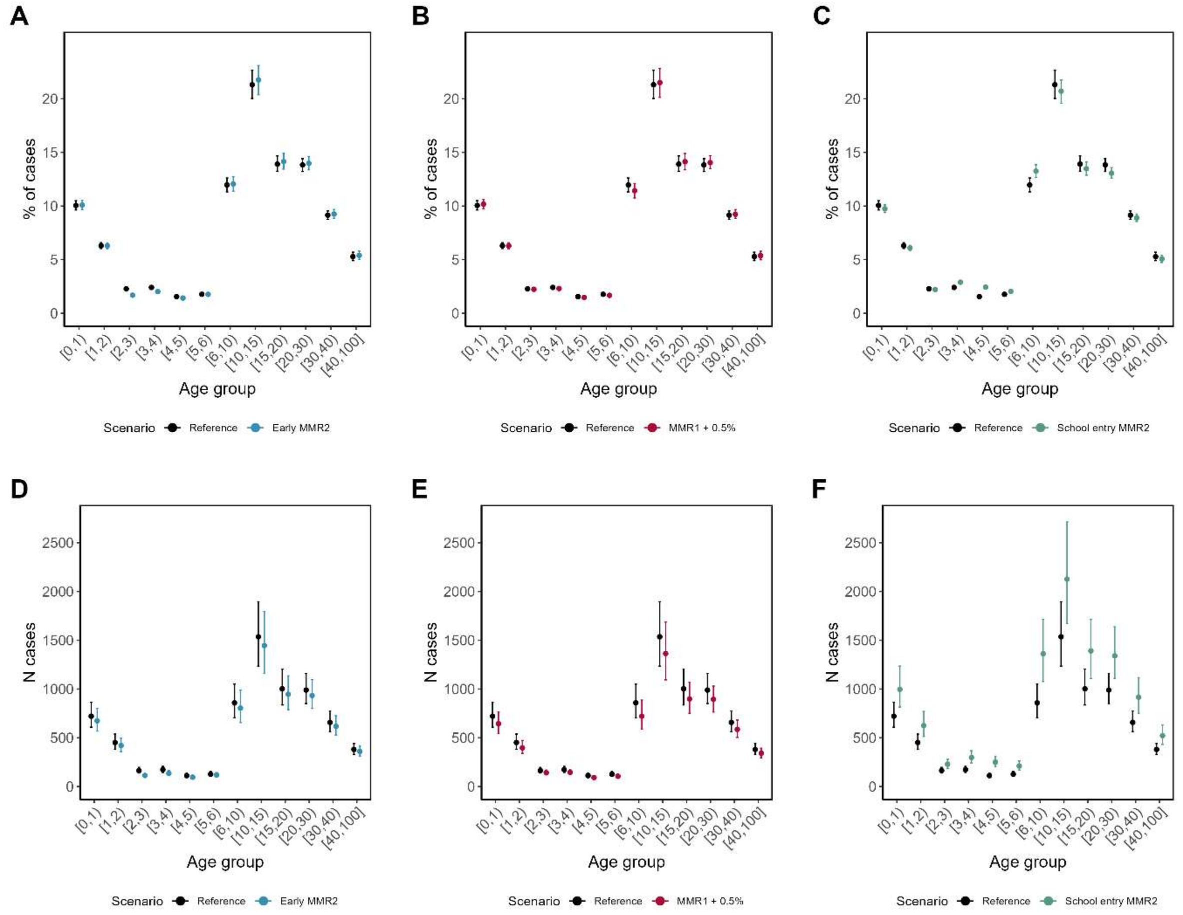

Figure S8: Age distribution of measles cases by different vaccination scenarios using COVER data. Comparison of measles cases per age compartment using COVER data as proportion of all cases and absolute numbers of cases between the reference scenario and an earlier MMR2 (A+D), between reference scenario and MMR1 improved by 0.5% (B+E) and MMR2 given at the age of five (C+F). The error bars represent the interquartile range across all 2500 simulations per scenarios.

S6. Impact of vaccine schedule and coverage on transmission dynamics: CPRD data with waning

Table S10.Number of measles cases and cases avoided by vaccination strategy using CPRD data and including waning since vaccination into the final model.

	**Waning from the age of five**		**Waning from the age of 3**	
**Vaccination Scenario**	**Median (IQR) number of cases across simulations**	**% of cases avoided in comparison to the median of the reference scenario (IQR)***	**Median (IQR) number of cases across simulations**	**% of cases avoided in comparison to the median of the reference scenario (IQR)***
**Reference**	7195 (6139.75; 8543)	0 (-18.74; 14.67)	-	-
Scenarios of different vaccination schedules				
**MMR1 +0.5%**	5945.5 (5098.75; 6936.5)	17.37 (3.59; 29.13)	-	-
**MMR1 +1%**	5063 (4399; 5858)	29.63 (18.58; 38.86)	-	-
Scenarios of different vaccination schedules				
**late MMR2**	8821.5 (7510.5; 10661.25)	-22.61 (-48.18; -4.38)	-	-
**early MMR2**	6815 (5782.75; 7980.5)	5.28 (-10.92; 19.63)	6922.5 (5840.25; 8125.5)	3.79 (-12.93; 18.83)
**Early MMR2 with coverage as MMR1**	6134.5 (5285.25; 7187.25)	14.74 (0.11; 26.54)	6248 (5360.75; 7214)	13.16 (-0.26; 25.49)
**Early MMR2 with decreased uptake - 3%**	6951 (5933.75; 8202)	3.39 (-14; 17.53)	7016.5 (5960.75; 8300.25)	2.48 (-15.36; 17.15)
**Early MMR2 with decreased uptake - 5%**	7060 (6015; 8455.25)	1.88 (-17.52; 16.4)	7114 (6083.75; 8435.5)	1.13 (-17.24; 15.44)

*A positive percentage is the percentage of cases avoided; a negative percentage is the percentage of additional cases in comparison to the median of the reference scenario. The proportion of the cases avoided in comparison to the median was calculated as follows: Ncases-Median(reference)/Median(reference))*100.

Table S11. Number of measles cases and cases avoided by vaccination strategy stratified by year for CPRD with waning from the age of 5

Year	Reference scenario	Early MMR2 – waning from age 5		Early MMR2 – waning from age 3	
	Median number of simulated cases (IQR)	Median number of simulated cases (IQR)	% change in comparison to the median of the reference scenario	Median number of simulated cases (IQR)	% change in comparison to the median of the reference scenario
2010	317 (222.75; 444.25)	307 (212; 428)	3.15 (-35.02; 33.12)	313 (218; 440)	1.26 (-38.8; 31.23)
2011	1389 (1112; 1788.25)	1352 (1076; 1729.25)	2.66 (-24.5; 22.53)	1361 (1076; 1732.25)	2.02 (-24.71; 22.53)
2012	1580 (1117.75; 2150.5)	1490 (1044.5; 2011.5)	5.7 (-27.31; 33.89)	1497.5 (1052.75; 2027)	5.22 (-28.29; 33.37)
2013	866.5 (548.75; 1280.25)	796 (498; 1181.25)	8.14 (-36.32; 42.53)	812.5 (509.75; 1198)	6.23 (-38.26; 41.17)
2014	515 (343; 762.25)	477 (317; 689.25)	7.38 (-33.83; 38.45)	479 (329.75; 702)	6.99 (-36.31; 35.97)
2015	303 (189; 486)	291 (180; 448)	3.96 (-47.85; 40.59)	295 (181; 446.25)	2.64 (-47.28; 40.26)
2016	262 (179; 378.25)	253 (170; 360)	3.44 (-37.4; 35.11)	248 (172; 366)	5.34 (-39.69; 34.35)
2017	342 (255; 452)	325 (239; 442)	4.97 (-29.24; 30.12)	333 (241.75; 448)	2.63 (-30.99; 29.31)
2018	560 (448.75; 677)	537 (431; 661.25)	4.11 (-18.08; 23.04)	539 (434; 668)	3.75 (-19.29; 22.5)
2019	670.5 (550; 802.5)	633 (524; 771)	5.59 (-14.99; 21.85)	658 (541; 791)	1.86 (-17.97; 19.31)

Table S12. Number of measles cases and cases avoided by vaccination strategy stratified by region for CPRD with waning from the age of 5.

**Region**	**Reference scenario**	**Early MMR2 – waning from age 5**		**Early MMR2 – waning from age 3**	
	**Median number of simulated cases (IQR)**	**Median number of simulated cases (IQR)**	**% change in comparison to the median of the reference scenario**	**Median number of simulated cases (IQR)**	**% change in comparison to the median of the reference scenario**
North East	157 (133; 187)	149 (127; 177)	5.10 (-12.74; 19.11)	150 (126; 179)	4.46 (-14.01; 19.75)
North West	729 (617; 865.5)	687 (581; 806)	5.76 (-10.56; 20.3)	698 (587; 825)	4.25 (-13.17; 19.48)
Yorkshire and the Humber	470 (396; 556)	443 (377; 518)	5.74 (-10.21; 19.79)	448 (378.75; 528.25)	4.68 (-12.39; 19.41)
East Midlands	504 (427; 606)	477 (401; 560)	5.36 (-11.11; 20.44)	487 (408; 572)	3.37 (-13.49; 19.05)
West Midlands	656 (555; 785.25)	623 (523; 729)	5.03 (-11.13; 20.27)	631 (531; 746)	3.81 (-13.72; 19.05)
East of England	749 (636; 895.25)	709 (603; 833)	5.34 (-11.21; 19.49)	719 (604; 847)	4.01 (-13.08; 19.36)
London	2077 (1770; 2455)	1967.5 (1689; 2310)	5.27 (-11.22; 18.68)	2009 (1693.75; 2352.25)	3.27 (-13.25; 18.45)
South East	1351 (1152; 1615)	1286 (1082; 1506)	4.81 (-11.47; 19.91)	1297.5 (1096.5; 1533.25)	3.96 (-13.49; 18.84)
South West	504 (428; 597)	476 (401; 558)	5.56 (-10.71; 20.44)	483 (407.75; 573.25)	4.17 (-13.74; 19.1)

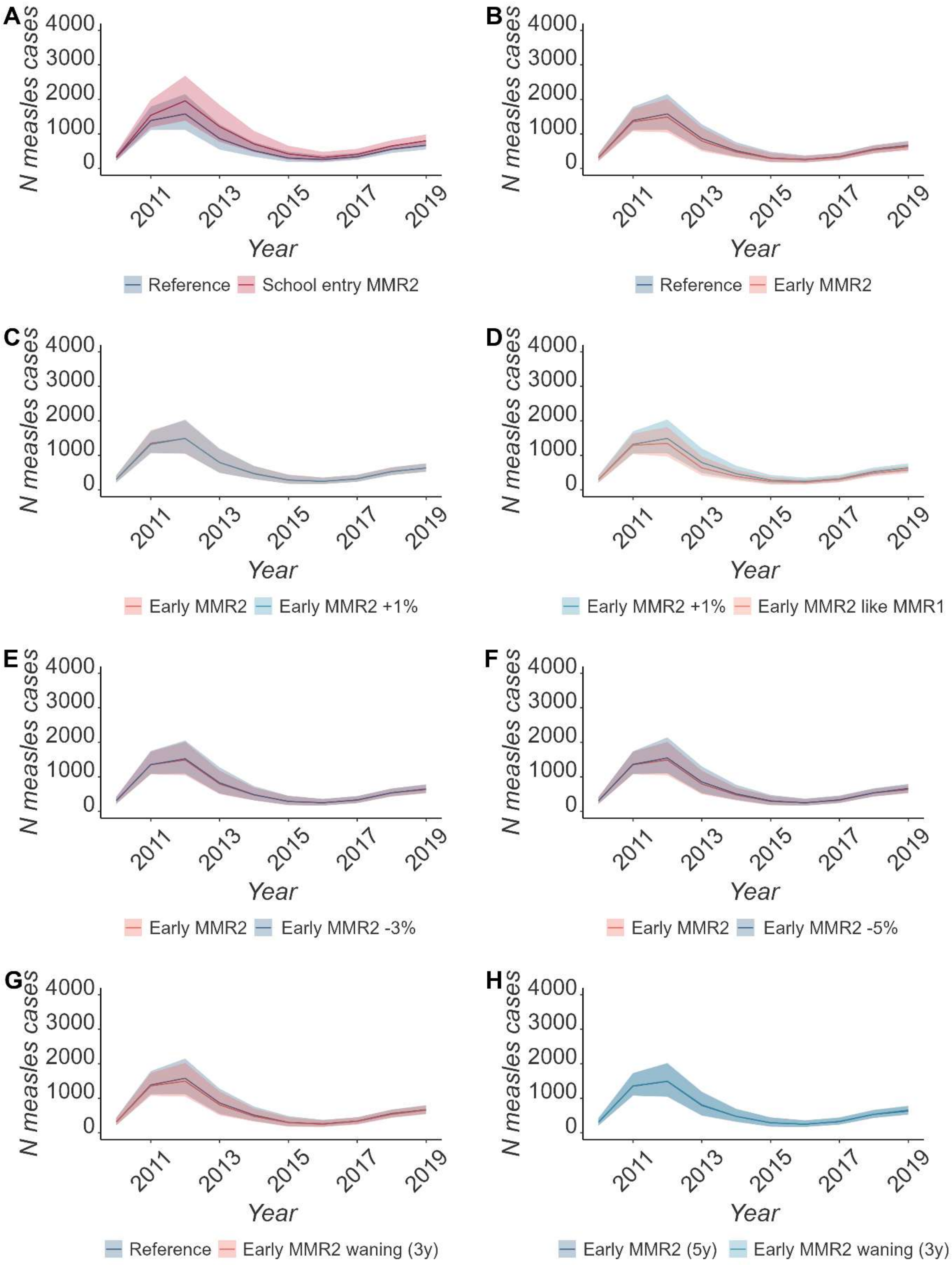

Figure S9: Comparing the reference scenario to scenarios with changes in the immunisation schedule including waning. Comparing the median number and IQR of simulated cases across simulations using CPRD data and including waning from the age of 5 between (A) Reference scenario and MM2 given at school age, (B) Reference scenario and MMR2 given at the age of two, (C) MMR2 given at the age two against an increase of

MMR1 by 1%., (D) increased MMR1 by 1% and an earlier MMR2 with the same coverage as MMR1. (E) and (F) are comparing the early MMR2 with the same uptake as before against a drop in coverage by 3% and 5% respectively. (G) compares the reference scenario to an early MMR2 when waning starts at age 3 and (H) compares an early MMR2 with waning from the age of 5 against an early MMR2 when waning starts at age 3.

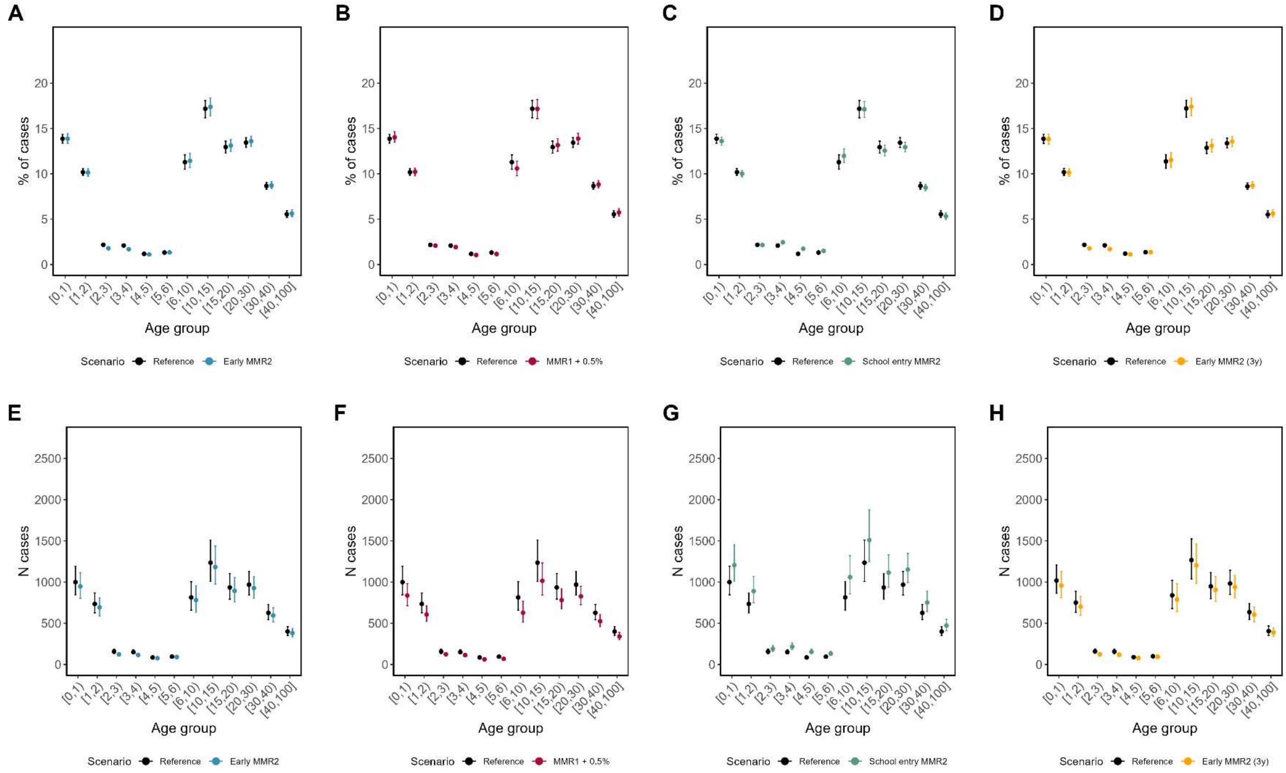

Figure S10: Age distribution of measles cases by different vaccination scenarios including waning. Comparison of measles cases per age compartment using the model with CPRD and waning from age 5 as proportion of all cases and absolute numbers of cases between the reference scenario and an earlier MMR2 (A+E), between reference scenario and MMR1 improved by 0.5% (B+F) and MMR2 given at the age of five (C+G), and the reference scenario against an earlier MMR2 when waning starts at age 3 (D+H). The error bars represent the interquartile range across all 2500 simulations per scenarios.

1. Robert A, Suffel AM, Kucharski AJ. Long-term waning of vaccine-induced immunity to measles in England: a mathematical modelling study. Lancet Public Health. 2024 Oct;9(10):e766–75.

2. FitzJohn RG, Knock ES, Whittles LK, Perez-Guzman PN, Bhatia S, Guntoro F, et al. Reproducible parallel inference and simulation of stochastic state space models using odin, dust, and mcstate. Wellcome Open Res. 2021 Jun 10;5:288.

3. Verguet S, Johri M, Morris SK, Gauvreau CL, Jha P, Jit M. Controlling measles using supplemental immunization activities: A mathematical model to inform optimal policy. Vaccine. 2015 Mar;33(10):1291–6.

4. Fu H, Abbas K, Klepac P, van Zandvoort K, Tanvir H, Portnoy A, et al. Effect of evidence updates on key determinants of measles vaccination impact: a DynaMICE modelling study in ten high-burden countries. BMC Med. 2021 Dec 17;19(1):281.

5. Mossong J, Hens N, Jit M, Beutels P, Auranen K, Mikolajczyk R, et al. Social Contacts and Mixing Patterns Relevant to the Spread of Infectious Diseases. Riley S, editor. PLoS Med [Internet]. 2008 Mar 25;5(3):e74. Available from: https://dx.plos.org/10.1371/journal.pmed.0050074

6. Guerra F, Crowcroft N, Friedman L, Deeks S, Halperin S, Severini A, et al. Waning of measles maternal antibody in infants in measles elimination settings - A systematic literature review. Vaccine. 2018;(Feb 28;36(10)):1248–55.

7. Rota JS, Hickman CJ, Sowers SB, Rota PA, Mercader S, Bellini WJ. Two Case Studies of Modified Measles in Vaccinated Physicians Exposed to Primary Measles Cases: High Risk of Infection But Low Risk of Transmission. J Infect Dis. 2011 Jul;204(suppl_1):S559–63.

8. UK Health Security Agency. 21 Measles. In: The Green Book [Internet]. 2019. Available from: https://www.gov.uk/government/publications/measles-the-green-book-chapter-21

9. Klinkenberg D, Nishiura H. The correlation between infectivity and incubation period of measles, estimated from households with two cases. J Theor Biol [Internet]. 2011 Sep;284(1):52–60. Available from: https://linkinghub.elsevier.com/retrieve/pii/S0022519311003146

10. European Centre for Disease Prevention and Control. Measles on the rise in the EU/EAA: considerations for public health response [Internet]. 2024 [cited 2025 Jan 9]. Available from:

https://www.ecdc.europa.eu/sites/default/files/documents/measles-eu-threat-assessment-brief-february-2024.pdf

42. Li, J. et al. Identification of the superconductivity in bilayer nickelate $La_3Ni_2O_7$ upon 100 GPa. *Natl. Sci. Rev.,* nwaf220 (2025). https://doi.org/10.1093/nsr/nwaf220.

43. Ko, E. et al. Signatures of ambient pressure superconductivity in thin film $La_3Ni_2O_7$. *Nature* **638**, 935-940 (2025).

44. Liu, Y. et al. Superconductivity and normal-state transport in compressively strained $La_2PrNi_2O_7$ thin films. *Nat. Mater.* (2025). https://doi.org/10.1038/s41563-025-02258-y
