# Supplementary material for: Impact of vaccination timing and coverage on measles near elimination dynamics: a mathematical modelling analysis
Source: Nat Commun. 2025 Sep 29;16:8601. doi: 10.1038/s41467-025-63710-w (PMC12480554; doi:10.1038/s41467-025-63710-w)
Supplement: Supplementary file 2 — Reporting Summary [file 41467_2025_63710_MOESM2_ESM.pdf]

nature portfolio

Corresponding author(s): Anne Marie Suffel

Last updated by author(s): Aug 12, 2025

Reporting Summary

Nature Portfolio wishes to improve the reproducibility of the work that we publish. This form provides structure for consistency and transparency in reporting. For further information on Nature Portfolio policies, see our Editorial Policies and the Editorial Policy Checklist.

Statistics

For all statistical analyses, confirm that the following items are present in the figure legend, table legend, main text, or Methods section.

n/a	Confirmed	
☐	☒	The exact sample size (*n*) for each experimental group/condition, given as a discrete number and unit of measurement
☐	☒	A statement on whether measurements were taken from distinct samples or whether the same sample was measured repeatedly
☒	☐	The statistical test(s) used AND whether they are one- or two-sided<br>*Only common tests should be described solely by name; describe more complex techniques in the Methods section.*
☒	☐	A description of all covariates tested
☐	☒	A description of any assumptions or corrections, such as tests of normality and adjustment for multiple comparisons
☐	☒	A full description of the statistical parameters including central tendency (e.g. means) or other basic estimates (e.g. regression coefficient) AND variation (e.g. standard deviation) or associated estimates of uncertainty (e.g. confidence intervals)
☒	☐	For null hypothesis testing, the test statistic (e.g. *F*, *t*, *r*) with confidence intervals, effect sizes, degrees of freedom and *P* value noted<br>*Give P values as exact values whenever suitable.*
☐	☒	For Bayesian analysis, information on the choice of priors and Markov chain Monte Carlo settings
☒	☐	For hierarchical and complex designs, identification of the appropriate level for tests and full reporting of outcomes
☒	☐	Estimates of effect sizes (e.g. Cohen's *d*, Pearson's *r*), indicating how they were calculated

Our web collection on statistics for biologists contains articles on many of the points above.

Software and code

Policy information about availability of computer code

Data collection	No primary data was collected.
Data analysis	All analyses were performed in R and R studio, version 4.2.2. The analysis code can be found in the following GitHub repository: https://github.com/Eyedeet/measles_vaccination_scenarios. The code has been published as version v1.0.0 (doi: 10.5281/zenodo.16812281) and used the v1.0.0 version of the alxsrobert/measles_england_sir package.

For manuscripts utilizing custom algorithms or software that are central to the research but not yet described in published literature, software must be made available to editors and reviewers. We strongly encourage code deposition in a community repository (e.g. GitHub). See the Nature Portfolio guidelines for submitting code & software for further information.

Data

Policy information about availability of data

All manuscripts must include a data availability statement. This statement should provide the following information, where applicable:

- Accession codes, unique identifiers, or web links for publicly available datasets
- A description of any restrictions on data availability
- For clinical datasets or third party data, please ensure that the statement adheres to our policy

The study uses data from the Clinical Practice Research Datalink (CPRD). CPRD does not allow the sharing of patient-level data. The data specification for the CPRD data set is available at: https://cprd.com/cprd-aurum-may-2022-dataset. The COVER data is publicly available: https://www.england.nhs.uk/statistics/statistical-

work-areas/child-immunisation/. The detailed outbreak data from PHE is not publicly available but summaries are shared online via NHS digital.
The analysis code can be found in the following GitHub repository: https://github.com/Eyedeet/measles_vaccination_scenarios and the generated scenarios and the simulations can be found in the repository (https://github.com/Eyedeet/measles_vaccination_scenarios/tree/main/Output)

Research involving human participants, their data, or biological material

Policy information about studies with human participants or human data. See also policy information about sex, gender (identity/presentation), and sexual orientation and race, ethnicity and racism.

Reporting on sex and gender	We don't report any analyses based on sex or gender.
Reporting on race, ethnicity, or other socially relevant groupings	We don't report any analyses based on race, ethnicity or other socially relevant groupings.
Population characteristics	We describe the population by age group (year of birth), and region of residence. Vaccination status was determined by health records in CPRD indicating a receipt of an MMR vaccine and report of vaccine coverage in COVER.
Recruitment	CPRD is collected anonymously across a network of GP practices around the UK but patients can withdraw their consent for the practice reporting their data. COVER is a quarterly data collection and compromises of aggregated GP data which is mandatory on a local-authority level. As measles are a notifiable disease, any case in England is reported to UKHSA when diagnosed.
Ethics oversight	We received data governance approval from CPRD (protocol number 22_001706) and ethical approval from the London School of Hygiene and Tropical Medicine's research ethics committee (reference number 27651).

Note that full information on the approval of the study protocol must also be provided in the manuscript.

Field-specific reporting

Please select the one below that is the best fit for your research. If you are not sure, read the appropriate sections before making your selection.

☒ Life sciences ☐ Behavioural & social sciences ☐ Ecological, evolutionary & environmental sciences

For a reference copy of the document with all sections, see nature.com/documents/nr-reporting-summary-flat.pdf

Life sciences study design

All studies must disclose on these points even when the disclosure is negative.

Sample size	We simulated measles outbreak for the entire English population based on estimates published by the Office of National Statistics. Sample size considerations do not apply. Vaccination estimates were obtained from CPRD as described in a previous publication cited in the manuscript and included data from 573,015 children in England at the age of five. COVER contains data of all children in England registered with a GP.
Data exclusions	N/A
Replication	We used to different estimates for vaccination coverage, one based on patient-level data from CPRD and COVER data published by the government. This aimed to ensure that our findings are robust to different methods of data collection. Both data sets showed similar trends with a slightly lower numbers of cases averted when using COVER data.
Randomization	This is a simulation study, randomization does not apply.
Blinding	This is a simulation study, blinding does not apply.

Reporting for specific materials, systems and methods

We require information from authors about some types of materials, experimental systems and methods used in many studies. Here, indicate whether each material, system or method listed is relevant to your study. If you are not sure if a list item applies to your research, read the appropriate section before selecting a response.

Materials & experimental systems

n/a	Involved in the study	
☒	☐	Antibodies
☒	☐	Eukaryotic cell lines
☒	☐	Palaeontology and archaeology
☒	☐	Animals and other organisms
☐	☒	Clinical data
☒	☐	Dual use research of concern
☒	☐	Plants

Methods

n/a	Involved in the study	
☒	☐	ChIP-seq
☒	☐	Flow cytometry
☒	☐	MRI-based neuroimaging

Clinical data

Policy information about clinical studies

All manuscripts should comply with the ICMJE guidelines for publication of clinical research and a completed CONSORT checklist must be included with all submissions.

Clinical trial registration	N/A
Study protocol	CPRD protocol number 22_001706
Data collection	pre-collected electronic health records
Outcomes	We used a combination of prescription codes and medical records from CPRD to determine vaccine uptake. The method has been validated and results including characteristics of the study population were published in the following paper: Suffel, AM., Walker, JL., Campbell, C., Carreira, H., Warren-Gash, C., & McDonald, HI. (2024). The methods applied where published here: A New Validated Approach for Identifying Childhood Immunizations in Electronic Health Records in the United Kingdom. Pharmacoepidemiology and Drug Safety, 33(8), Article e5848. https://doi.org/10.1002/pds.5848

Plants

Seed stocks	n/a
Novel plant genotypes	n/a
Authentication	n/a
